# P300 correlates with learning & memory abilities and fluid intelligence

**DOI:** 10.1186/s12984-015-0077-6

**Published:** 2015-09-23

**Authors:** Hafeez Ullah Amin, Aamir Saeed Malik, Nidal Kamel, Weng-Tink Chooi, Muhammad Hussain

**Affiliations:** Centre for Intelligent Signal & Imaging Research (CISIR), Department of Electrical & Electronic Engineering, Universiti Teknologi PETRONAS, 32610 Bandar Seri Iskandar, Perak Malaysia; Advanced Medical and Dental Institute (AMDI), Universiti Sains Malaysia, 11900 Gelugor, Penang Malaysia; Department of Computer Science, College of Computer and Information Sciences, King Saud University, 12372 Riyadh, Saudi Arabia

**Keywords:** Event related potentials (ERPs), P300, Fluid intelligence, Learning and memory recall

## Abstract

**Background:**

Educational psychology research has linked fluid intelligence with learning and memory abilities and neuroimaging studies have specifically associated fluid intelligence with event related potentials (ERPs). The objective of this study is to find the relationship of ERPs with learning and memory recall and predict the memory recall score using P300 (P3) component.

**Method:**

A sample of thirty-four healthy subjects between twenty and thirty years of age was selected to perform three tasks: (1) Raven’s Advanced Progressive Matrices (RAPM) test to assess fluid intelligence; (2) learning and memory task to assess learning ability and memory recall; and (3) the visual oddball task to assess brain-evoked potentials. These subjects were divided into High Ability (HA) and Low Ability (LA) groups based on their RAPM scores. A multiple regression analysis was used to predict the learning & memory recall and fluid intelligence using P3 amplitude and latency.

**Results:**

Behavioral results demonstrated that the HA group learned and recalled 10.89 % more information than did the LA group. ERP results clearly showed that the P3 amplitude of the HA group was relatively larger than that observed in the LA group for both the central and parietal regions of the cerebrum; particularly during the 300–400 ms time window. In addition, a shorter latency for the P3 component was observed at Pz site for the HA group compared to the LA group. These findings agree with previous educational psychology and neuroimaging studies which reported an association between ERPs and fluid intelligence as well as learning performance.

**Conclusion:**

These results also suggest that the P3 component is associated with individual differences in learning and memory recall and further indicate that P3 amplitude might be used as a supporting factor in standard psychometric tests to assess an individual’s learning & memory recall ability; particularly in educational institutions to aid in the predictability of academic skills.

## Introduction

The assessment of academic learning performance remains a common practice in education to support the decisions related to student selection. Presently, academic performance metrics are used to support decisions associated with grading, judgment, selection and placement. It is therefore assumed that earlier assessment of an individual’s ability to both learn and recall knowledge would improve learning management strategies and interventions.

Cognitive ability, or fluid intelligence, has been commonly used to help predict an individual’s capacity and ability for academic learning [1–3]. The assessment of fluid intelligence involves the use of deliberate mental operations that are employed to solve novel problems that cannot be accomplished by simple memorization [4]. Furthermore, several cognitive psychological studies have associated fluid intelligence with learning ability as predictors of an individual’s learning capacity and ability [2, 3, 5, 6] and results from such studies have potential implications for learning practices. However, measurement of cognitive capabilities using intelligence testing has many limitations, e.g., it covers only on verbal and mathematical skills, and it is time consuming as well.

Recent neurophysiological studies investigated individual variation in different cognitive processes including information processing, working memory, and intelligence by measuring event-related brain potentials (ERPs) [7, 8]. ERPs represent time-locked voltage fluctuations in electroencephalographic (EEG) recordings that are demonstrably sensitive to cognitive events and have been widely adopted to analyze event-related EEGs [8–10]. One of most frequently reported ERP component is the P300 (also referred to as ‘P3’) in previous studies, particularly in information processing [8]. It is closely related to attentional resource allocation and working memory in both frontal and parietal regions of the brain [9]. P3 is extracted from ERP signals between 250–500 ms after stimulus onset—the range may vary depending on stimulus modality, task conditions, subject age, etc., [8]. The P3 latency component is considered a direct indicator of a subject’s stimulus evaluation and speed of information processing; thus, it is taken as a metric representing the strength of cognitive processing [11].

A number of studies have associated P3 amplitude and latency with levels of intelligence [7], speed of information processing [11], executive function [12], and stimulus change detection [8]. P3 latency has been reported to be inversely related to the level of a person’s intellectual ability [7, 12], which may then infer a positive relationship between intelligence and the mental speed of information processing. Nevertheless, the relation of P3 with intelligence remains unclear. There are, however, some studies that claim a positive correlation between P3 amplitude and intelligence [7, 13] while several others have reported a negative or zero correlation [14, 15]. Possible reasons for the contradiction were recently proposed by Wronka et al. [7]. These include (i) a positive correlation due to memory related tasks; and (ii) a negative correlation when perceptual tasks or stimuli detection tasks initiate two different sets of cognitive processes [7].

Based on our literature review, fluid intelligence bear a noticeable correlation with the P3 component as well as learning and memory recall abilities [4, 6, 7, 13, 16]. Hence, studies reporting variations in P3 amplitude and latency for midline electrodes among high and low cognitive ability individuals were taken as the foundation for the present effort [7, 9, 17]. We hypothesized: (i) that high cognitive ability (HA) subjects would show ‘relative increase’ in P3 amplitude at centro-parietal loci, bearing high learning & memory ability; and (ii) that low cognitive ability (LA) subjects would show ‘relative decrease’ in P3 amplitude at centro-parietal loci, with low learning & memory ability.

In view of the stated hypotheses, the present study attempted to associate ERPs with learning and memory recall as well as fluid intelligence. A sample of thirty-four healthy subjects between twenty and thirty years of age was recruited to perform three experimental tasks: (1) Raven’s Advanced Progressive Matrices (RAPM) test to assess fluid intelligence; (2) learning and memory tasks to assess learning ability and memory recall; and (3) the visual oddball task to assess brain-evoked potentials. On the basis of RAPM scores, the subjects were divided into two groups, (i) High ability (HA) group―subjects scored above the median; and (ii) Low ability (LA) group―subjects scored equaled or scored below the median. Such division of subjects on the median value of intelligence test was previously reported by Wronka et al. [7]. ERPs were extracted from the EEG recordings of thirty-four subjects as recorded while they undertook a visual oddball task which presented Standard (box) and Target (sphere) stimuli. Each subject’s target and standard stimuli responses were averaged individually from which a collective response average was then calculated. The focus of this paper is on P3 component only. However, to investigate whether the differences between the two groups are specific to P3 only, the P200 (P2) component was also extracted and considered for analysis. The amplitude and latency of both P2 & P3 components were performed on the difference between target and standard responses from 20 electrodes (19 electrodes based on 10–20 system with additional Oz electrode). The difference of target and standard would show more strongly the P3 response at parietal regions and would cancel the ERP elicited by target or standard stimuli at frontal and central regions. In addition, the grand averaged target and standard waveforms as well as their respective difference of both groups with topographical variations from frontal regions to fronto-central, centro-parietal, and occipital regions were also observed and recorded. These variations in the P3 component reflected and described individual differences in learning and memory recall as well as fluid intelligence. This study’s contribution indicates that the P3 component may be a reliable adjunct to standard psychometric tests which are used to predict a person’s ability to learn new knowledge and recall memories.

The paper is organized as follows: Methods and materials describes details of the experimental set-up, data recording and analysis; Results presents our results and discussion; Discussion concludes the paper.

## Methods and materials

### Subjects

A sample of 34 ostensibly healthy university students (all male; 31 right handed; 03 left handed; ranging from 20–30 years of age) was recruited for the experiment. They had normal or ‘corrected to normal’ vision and were free from medication, neurological disorders and hearing impairments. All signed an informed consent document prior to beginning the trials. This study was approved by the Ethics Coordination Committee of the Universiti Teknologi PETRONAS, and by the Human Research Ethics Committee of the Universiti Sains Malaysia.

### Raven’s Advanced Progressive Matrix (RAPM) test

**Fig. 1:**
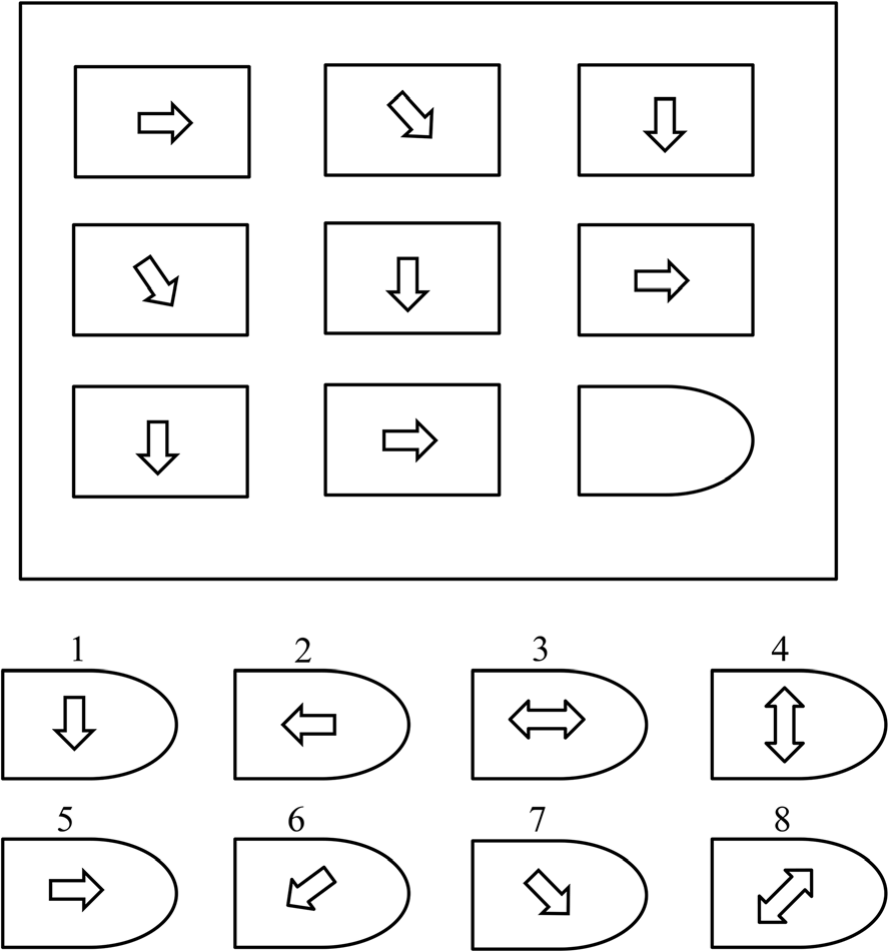
A simple Raven’s style pattern (Option no. 7 is correct answer for this pattern)

Raven’s Advanced Progressive Matrix (RAPM) [18] is a non-verbal test used to measure intellectual ability. It commonly and directly measures two components of fluid cognitive ability [18] defined as: (i) “The ability to draw meaning out of confusion; and (ii) the ability to recall and reproduce information that has been made explicit and communicated from one to another.” It comprises a series of 48 patterns divided into two sets (I & II). Set-I contains 12 patterns used for practice; Set-II contains 36 patterns used to assess cognitive ability. Each pattern contains a 3×3-cell structure in which each cell represents a certain geometrical shape excepting the right-bottom cell which is empty as shown in Fig. 1. Eight multiple options are given for the empty cell. A score of ‘1’ is assigned for each correct answer and a score of ‘0’ for an incorrect answer. Recommended administration time was used, i.e., 10 min for Set-I and 40 min for Set-II [18, 19].

### Learning & memory recall tasks

Previous studies have used simple stimuli for learning and memory tasks such as color images [20], digits [21], words and pictures [22], video clips [23], and associative learning tasks with artificial words [24]. However, the learning material used for this study was based on biology (human anatomy). Commercially available, high quality computer animations of biological subjects used for standard high secondary curriculum (grades 11–12) were used as the learning content (see: *Designmate* at www.designmate.com). A total of 8 ~ 10 min. of content were selected related to complex human anatomy concepts, functions and diseases. The subjects had no prior knowledge of the learning material and most had backgrounds in engineering and mathematics. Hence, this select learning content provided new information suitable for the assessment of learning and memory skills. In addition to the learning session, a memory recall test was prepared consisting of twenty multiple-choice questions (MCQs) covering the newly learned material. Each MCQ comprised of a brief question statement with four options as possible correct answers. Subjects were given 30 s to answer each MCQ within a maximum limit of 10 min total. They were asked to press a numeric key on the keyboard, serially numbered #1 to #4 corresponding to each possible answer. An example of MCQ is given in the following box.

**Table:** 

*Q. The damage of epithelial wall activates the platelets to form______.*	
**1.** *Fibrin thread*	
**2.** *Good cholesterol*	
**3.** *HDL*	
***4.*** *Clot*	

### Visual oddball task

The visual oddball task is commonly used for ERP studies. Here, visual stimuli are presented to assess neural activity during cognitive and attention demanding events [8]. All subjects performed the visual oddball task where box and sphere shapes were used as standard and target stimuli, respectively. The size of both standard and target stimulus was the same (5 cm). For each trial, a standard (box) or target (sphere) stimulus was presented for 500 ms with an inter-trial-interval (ITI) of 500 ms between trials. The task required subjects to press “0” when a target stimulus appeared and ‘not to respond’ for a standard stimulus. Subjects were instructed to respond as quickly as possible while avoiding errors. Reaction time and correct target detection were recorded. The task contained 135 trials, in which 40 trials contained target stimulus and 95 trials contained standard stimulus (i.e., 30 % of the trials contained a target stimulus and 70 % contained a standard stimulus). The duration of the task was approximately 4 min. This task was modified according to [25].

### Experiment procedure

All subjects were informed of the schedule for data collection and, as per their availability, experiments were arranged individually. Before going to perform the actual experiment, each subject was asked to solve a list of 10 descriptive questions related to the experimental learning contents as a pre-test for controlling the background knowledge. The exclusion criteria were 10 % i.e., at-most one correct answer was allowed or otherwise ‘exclude subject’. However, no subject showed previous background knowledge about the learning contents used in this experiment and HA and LA groups were balanced.

Each subject was seated in a partially sound-attenuated room and briefed on the procedure. Each subject was asked to perform the RAPM first, after which learning material was presented twice. At the end of the learning session, a thirty-minute waiting period ensued before testing the subject’s retention. During this time, an EEG cap was set, as per procedure, and subjects were asked to perform the visual oddball task, which lasted about four minutes. A memory recall test then followed for each subject to assess learning and memory performance. Each RAPM and learning task was presented on a ‘41’ inch TV screen at a distance of 1.5 m from the subject. All tasks were implemented with the E-Prime Professional 2.0 (Psychology Software Tools, Inc., Sharpsburg, PA) [26].

### Electrophysiological recordings

**Fig. 2:**
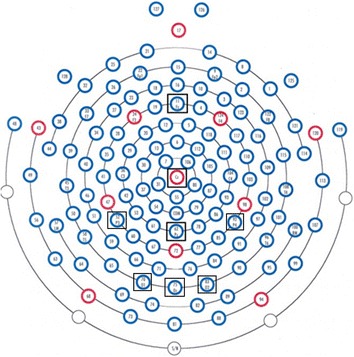
Electrode placement (HydroCel Geodesic Net)

The EEG continuously recorded subject responses via 128 scalp loci using the HydroCel Geodesic Sensor Net (Electrical Geodesic Inc., Eugene, OR, USA) for the oddball task (see, Fig. 2). All electrodes referenced a single vertex electrode, Cz, from which raw signals were amplified with the EGI NetAmps 300 amplifier’s band pass filter (0.1–100 Hz). Impedance was maintained below 50KΩ and the sampling rate was 250 Hz.

#### Preprocessing

After the recording of raw EEG data, each subject’s continuous EEG data was preprocessed with NetStation v4.5.4 (Electrical Geodesic, Inc. Eugene, OR, USA). A brief description of the preprocessing and ERP extraction is provided here.

- A band pass filter was applied (0.3-30Hz, roll off 12 dB octave) to remove DC components and high frequency muscular artifacts.
- Next, each individual EEG trial was segmented by using a 600 ms window that comprised a 100 ms pre-stimulus period as a baseline followed by a 500 ms post-stimulus period.
- Individual trials were rejected, if containing artifacts (eye blinks and eye movements) i.e., the EEG amplitude exceeded maximum amplitude of ±90 μV in any segment was excluded.
- All segments were manually visualized and contributions were rejected from electrodes that had lost contact in the event of widespread drift [27]. Bad channels were discarded from the segments before averaging using spherical spline method [28].
- Subsequently, individual averaged waveforms were computed for each experimental condition (target and standard). Only good segments were retained in the individual averaged waveforms for target and standard condition, respectively, after artifact rejection.
- Data were then re-referenced from a single vertex (Cz) to the averaged reference. Finally, the difference between the target and standard waveforms was computed for each individual subject as well as for grand averaged of HA and LA groups. The ERP analysis was performed on the difference of target and standard responses.

ERP analyses were normally performed on midline electrodes (Fz, Cz, Pz) in previous studies [10, 29]. However, in this study, 20 electrodes were selected based on 10–20 International System of electrode placement with additional Oz electrode from 128 electrodes.

### Data analysis

#### Behavioral analysis

Behavioral data were analyzed to measure performances corresponding to fluid cognitive, learning and memory recall abilities as well as the visual oddball task.

To assess fluid cognitive ability, RAPM raw scores were calculated for each subject. The score range was 0–36 with a median score for all subjects of 24.50 and mean score of 23.57 (SD = ±5.6). On the basis of respective RAPM median scores, subjects who scored above the median were placed in the high ability (*HA*) group and those who equaled or scored below the median were placed in the low ability (*LA*) group [7]. Accordingly, sixteen subjects were placed in the HA group and fourteen subjects were placed in the LA group.

For learning and memory performance assessment, correct responses and reaction times were calculated per question for each subject. Reaction time, which reflects the mental speed of information processing, was measured from the point of multiple choice question (MCQ) display until the subject selected an answer. The percentage of correct responses per subject was then used to measure his/her learning performance.

For the visual oddball task, each subject’s performance ability metrics were computed by calculating the number of correctly detected target stimuli in addition to reaction time.

#### ERP analysis

For ERP analysis, both ERP amplitude and latency components were extracted from the respective 20 electrodes for each subject per group.

*Amplitude*(*S*_*max*_)― Is the maximum signal value at some point in time for a specified window [30]. Time window for P2 and P3 were 150–275 ms and 276–500 ms, respectively. The amplitude of both P2 and P3 components for a single trial was calculated as follows:

1 $$ {s}_{max,i}=ma{x}_t\left\{s(t)\Big|{w}_i(t)\right\} $$

where, *w*(*t*) = {150*ms* ≤ *t* ≤ 275*ms*, 276*ms* ≤ *t* ≤ 500*ms*}

Each subject’s average amplitude was calculated for all N trials of the oddball task.

The average amplitude or each group (HA and LA) was calculated for all subjects in each group, respectively.

*Latency*(ts_max_)― The latency of an ERP component is that point in time where the maximum signal value occurs [30]. The latency of a single trial was specified as follows:

2 $$ t{s}_{max}=\left\{t\Big|s(t)={S}_{max}\right\} $$

where s(t) is the ERP signal for a single trial at time *t* after stimulus onset; and s_max_ is the maximum signal value in a specified time window. For average latency, *ts*_*max*_ was averaged for all trials per subject. The average latency for each group (HA and LA) was calculated for all subjects in each group.

#### Feature ranking & selection

A total of 20 electrodes were included in the ERP analysis which were further reduced to 8 electrodes by using Fisher’s discriminant ratio (FDR) for statistical analysis including correlation and regression analysis. The FDR ranked all the features according to their discrimination power and independent of the type of class/group distribution. The FDR of a feature in two groups can be defined as following.

3 $$ \mathrm{F}\mathrm{D}\mathrm{R}=\frac{\left({\mathrm{m}}_1-{\mathrm{m}}_2\right)}{\left({\upsigma}_1^2-{\upsigma}_2^2\right)} $$

Where, *m*_*1*_ and *m*_*2*_ are mean values and *σ*_1_^2^ and *σ*_2_^2^ are the respective variances of a feature *x*_*i*_ in two groups.

#### Statistical analysis

A one way multivariate analysis of variance (MANOVA) was used to examine significant differences in the behavioral responses (accuracy in oddball task and accuracy in memory recall task) between both groups. The amplitudes and latencies of P2 and P3 components for differences between groups were analyzed using MANOVA with electrodes as a factor. Similarly, P3 amplitude & latency and memory recall score were treated as dependent variables and one way MANOVA was used to identify significant differences between groups. For overall relationship of ERPs with learning & memory ability as well as fluid intelligence, bivariate correlation analysis was employed for P3 amplitude, P3 latency, RAPM scores, and memory recall score from all the subjects. Pearson’s correlation for multiple comparisons was performed with Bonferroni correction to identify the association of different scalp site (midlines, parietal and occipital sites) with memory recall in the P3 analysis. A multiple regression analysis was applied to predict the memory recall from P3 amplitude and latency values. Furthermore, Cohen’s *d* was derived to show the effect size for (i) HA and LA groups for ERP parameters (amplitude and latency) as extracted from different electrode sites; and (ii) for both behavioral test scores: i.e., RAPM score as well as memory recall score.

### Multiple linear regression model

Multiple linear regression (MLR) is a linear statistical method that is used for predicting the relationship of a single dependent variable (response variable: *Y*) with one or more independent variables (predictors: *X*_1_, *X*_2_, …, *X*_*n*_) [31]. A general MLR model can be accomplished by the following equation:

4 $$ Y={\beta}_0+{\beta}_1{X}_1+\dots +{\beta}_n{X}_n+\varepsilon $$

Where Y represents the dependent variable, *X*_*i*_ indicates the *i*^*th*^ independent variable, *β*_*i*_ represents *i*^*th*^ predicted parameter (regression weight), and ε is the error between predicted response and the observation. The regression weights are computed in such a way that minimizes the sum of squared deviations.

In this study, MLR analysis was carried out by using SPSS 20.0 (Statistical Package for the Social Science) with stepwise method on the selected electrodes to predict the leaning & memory ability (Y_1_:memory recall) and fluid intelligence (Y_2_: RAPM score). The dependent variables and independent variables for the above equation are as follows:

- ▪ Dependent variables (Y_1_ = Memory recall, Y_2_ = RAPM score)
- ▪ Independent variables (amplitudes: X_1_ = Pz, *X*_2_ = P4, X_3_ = P3, X_4_ = O1, X_5_ = O2, X_6_ = Oz, X_7_ = Fz, X_8_ = Cz and from X_9_ to X_16_ represents the latencies of selected electrodes)

In order to evaluate the linear regression model statistically, the following important assumptions about the residuals were considered and verified [31].

1. The residuals should have zero mean value.
2. The residuals should be plotted as normal distribution.
3. The residuals should have constant variance (homoscedasticity).
4. The residuals are independent (or random).

The assumption (1) is easily verified and the rest of the assumptions are checked via plots of standardized residuals. If a normal probability plot of the standardized residuals will show straight line then assumption (2) is correct. The assumptions (3) and (4) can be evaluated by using the scatter plots which show the relationship between standardized residuals and the predicted values. The verification of these assumptions is given in section Verification of regression assumptions.

#### Performance evaluation of MLR model

To evaluate the predictive capability of the regression model, R^2^ (observed squared correlation coefficient) and R_cv_^2^ (cross-validated squared correlation coefficient) is used. R^2^ is a fraction between 0 and 1 (0 ≤ R^2^ ≤ 1) and interpreted as ‘no relationship between dependent and independent variables, if R^2^ = 0’ and ‘perfect relationship between dependent and independent variables, if R^2^ = 1.

The R_cv_^2^ is based on the leave-one-out (LOO) cross validation method which repeats the regression model N times, where N is the number of samples. Each time exactly N-1 samples are utilized to build the model and remaining one is used for prediction.

Receiver operating characteristic (ROC) technique was adopted for evaluation of MRL model (for more detail about ROC technique see [32]). The diagnostic accuracy of the ROC curve is the area under the curve (AUC). The value of AUC closer to 1 indicates perfect diagnostic accuracy.

## Results

There were a total of 135 trials in the oddball task, in which 40 trials contained target stimulus and 95 trials contained standard stimulus. Subjects were excluded from further ERP analysis due to an insufficient number of target segments (less than 20 good target segments out of 40 segments) that failed to obtain adequate ‘signal to noise ratio’. This exclusion allowed thirty subjects for final analysis and excluded four subjects.

The grand averaged waveforms of HA subjects contained an average of 30.56 and 83.00 good segments for target and standard condition, respectively; while the grand averaged waveforms of LA subjects contained an average of 26.64 and 76.14 good segments for target and standard condition, respectively. The mean age of HA and LA was 24.08 (SD = ±3.35) and 24.80 (SD = ±2.53) years and mean education (number of years completed) was 14.88 (SD = ±2.06) and 15.71 (SD = ±1.49), respectively. There were no statistical significant differences (*t*-test *p*-value > 0.05) between HA and LA for age and education.

### Behavioral results

Behavioral data recorded during the oddball and memory recall task were analyzed for both groups (HA and LA). A separate one way MANOVA was performed on the accuracy (ACC), F(2,27) = 5.34, *p* = 0.011, Wilk’s Λ = 0.716, partial η^2^ = .28; as well as on the reaction time (RT), F(2,27) = 3.86, *p* = 0.034, Wilk’s Λ = 0.778, partial η^2^ = .22, in the oddball task and memory recall task. Univariate analysis indicated significant differences in the ACC of oddball task, F(1,28) = 4.34; *p* = 0.046, as well as memory recall task, F(1,28) = 8.10; *p* = 0.008. Again, univariate analysis for RT in memory recall task was significant, F(1,28) = 7.23, *p* = 0.012; but not in the oddball task, F(1,28) = 0.88, *p* = 0.356. Additionally, Cohen’s d results (Table 1), indicated an intermediate to substantial effect size between HA and LA group performances. These results clearly indicated that the HA group’s performance was significantly higher than the LA group’s performance for both memory recall task and the oddball task. The mean percentage of accuracy (ACC) and mean reaction time (RT) of all the tasks are presented in Table 1 for both groups.

**Table 1:** Performance measurements

Group	Oddball task		RAPM		Memory recall task	
	ACC%	RT (ms)	ACC%	RT (s)	ACC%	RT (s)
HA	79.84(11.2)	426.20(22.8)	77.43(6.7)	47.80(19.9)	83.75(8.2)	7.75(2.1)
LA	70.71(12.8)	432.92(14.8)	51.79(11.2)	48.31(19.1)	72.86(12.5)	10.21(2.8)
^*^Effect Size (Cohen’s d)	0.79	0.36	2.93	0.03	1.08	1.04

Note: results are organized as mean plus (standard deviation) in 3^rd^ and 4^th^ row

^*^Small Effect 0.15 ≤ d < 0.40; Medium Effect 0.40 ≤ d < 0.75; Large Effect 0.75 ≤ d < 1.10; Very Large Effect 1.10 ≤ d < 1.45, Huge Effect d > 1.45

These behavioral results supported our hypothesis as we expected high performances in memory recall task as well as in oddball task from the HA group; and comparatively low performances from the LA group.

### ERP results

The ERP features extracted from the 20 electrodes were reduced to 8 electrodes/features based on their amplitude values using FDR. The FDR power discrimination value for parietal sites (Pz, P4, and P3), occipital (Oz, O2, and O1) sites, Fz and Cz were 0.93, 0.19, 0.12, 0.09, 0.08, 0.03, 0.02 and 0.009 respectively. Further, these selected electrodes (see Fig. 2) were used for identifying the differences between HA and LA groups and correlation with learning & memory ability (memory recall score) as well as fluid intelligence (RAPM score).

**Fig. 3:**
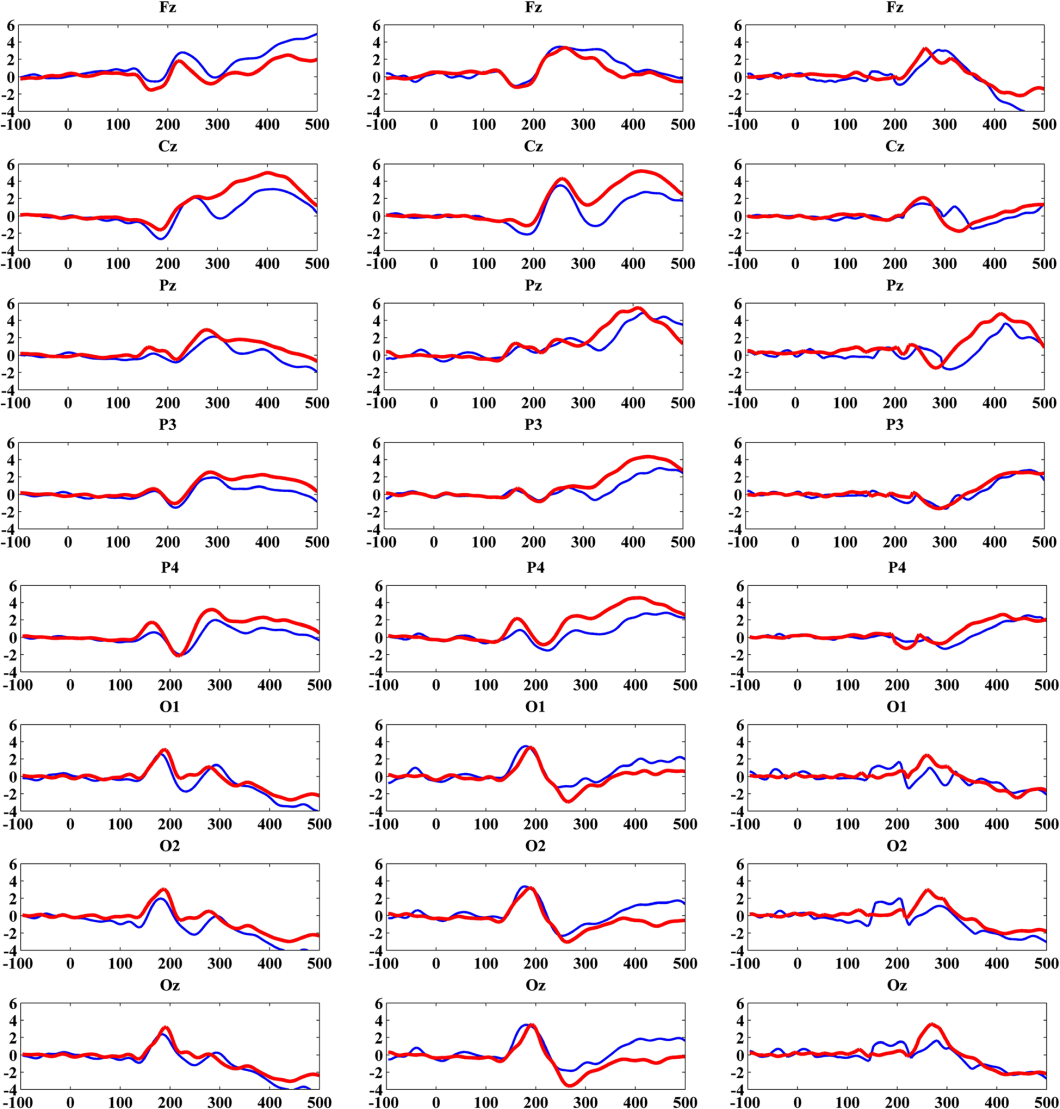
Average ERP waveforms of LA (blue) and HA (red) Groups (Left column represents the standard responses, middle column represents target response and right column represents difference between target and standard responses)

**Fig. 4:**
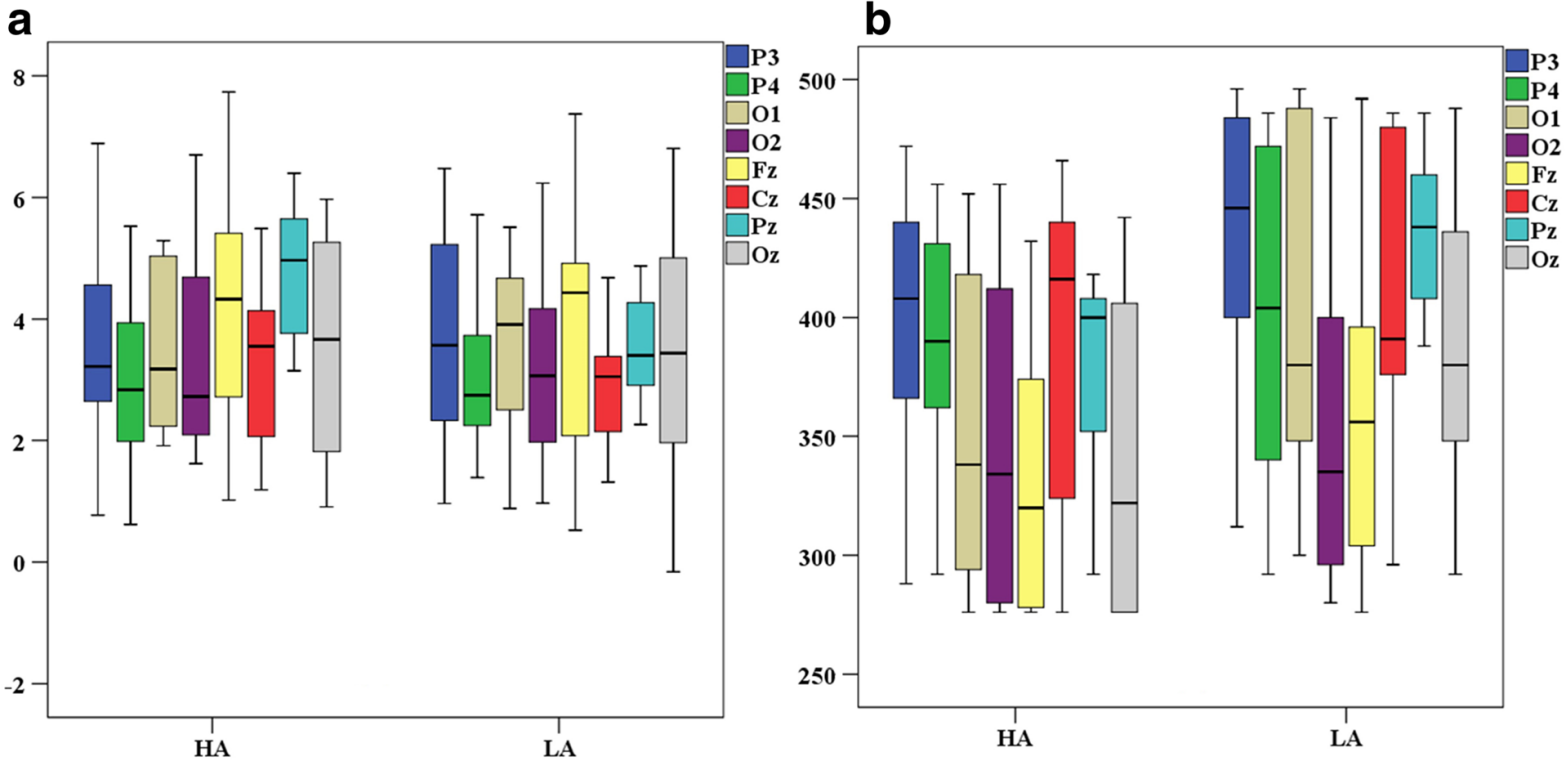
Box plots of the difference between Target and Standard responses (**a**) P3 amplitude and (**b**) P3 latency distribution

The overall averaged ERP waveforms evoked by standard, target and difference of standard and target stimuli in the oddball task for both HA and LA groups are shown in Fig. 3. The ERP components P2 and P3 were analyzed for time window 150-275 ms and 276–500 ms at the selected electrodes (see Fig. 4 for distribution of P3 amplitude and latency). P3 amplitude showed statistically significant differences between HA and LA groups, F(8,21) = 4.767, Wilk’s Λ = 0.355, *p* = 0.002, partial η^2^ = 0.645; particularly at Pz site, F(1,28) = 19.53; *p*-value = 0.0005. Similarly, P3 latency also showed statistically significant differences between HA and LA groups, F(8,21) = 2.53, Wilk’s Λ = 0.509, *p* = 0.042, partial η^2^ = 0.491, especially at Pz, F(1,28) = 13.672, *p*-value = 0.001. However, the P3 amplitude and latency were not significant at other sites such as Fz, Cz, and Oz (see Table 3). There was no significant difference in amplitude or latency of P2 component between groups indicated by the univariate analysis of variance i.e., the *p*-value > 0.05 for amplitude and latency in all the selected electrodes.

### Correlation results

**Fig. 5:**
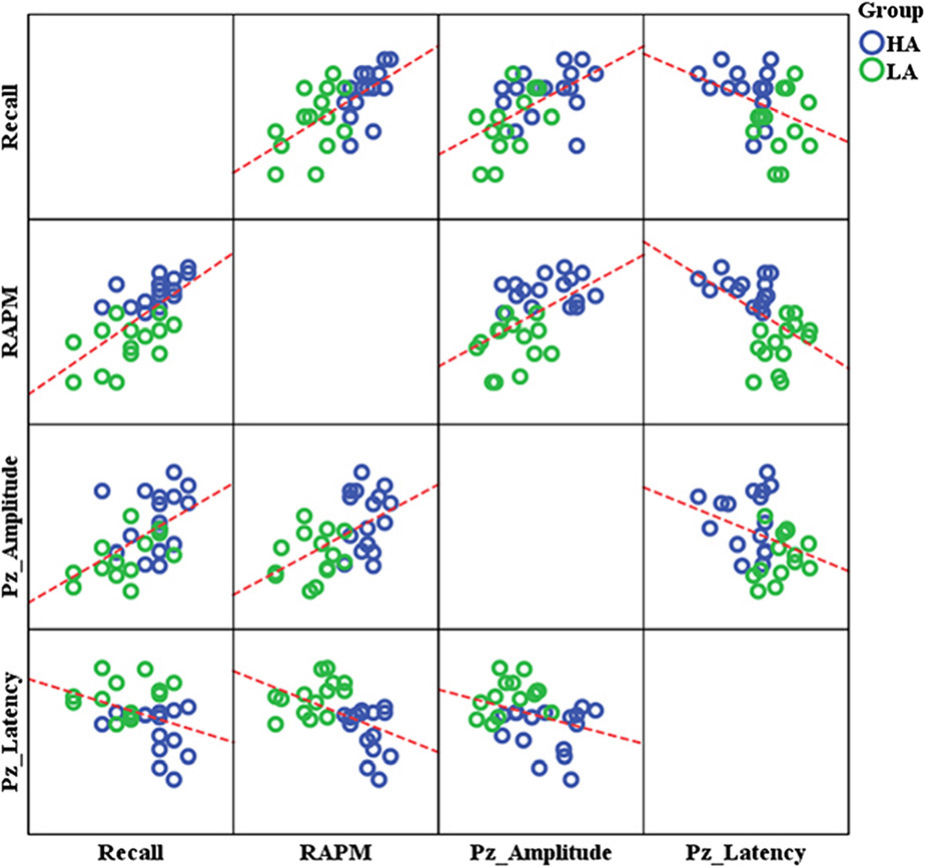
Scatter Plots represent the relationship of learning & memory (Recall), Cognitive Ability (RAPM), P3 Amplitude (APz), and P3 Latency (LPz). (Recall; RAPM:R^2^ = 0.427, Recall; APz: R^2^ = 0.307, Recall; LPz:R^2^ = 0.133, RAPM; APz: R^2^ = 0.291, RAPM; LPz:R^2^ = 0.245, APz; LPz: R^2^ = 0.107)

Bivariate correlation results showed that relationships existed between P3 component for both RAPM as well as memory recall scores (see Table 2 and Fig. 5). Interestingly, the correlation between the P3 amplitude at the parietal site and memory recall was r = 0.554 with *p*-value = 0.001, showing a significant relationship which is not previously reported.

**Table 2:** Bivariate correlation between P3 component (amplitude & latency), RAPM, and Memory recall (Pearson’s correlation coefficient)

Variables	Memory recall	RAPM	P3 amplitude (Pz)	P3 latency (Pz)
Memory Recall		0.653^***^	0.554^***^	−0.365^*^
RAPM	0.653^***^		0.540^***^	−0.495^**^
P3 amplitude (Pz)	0. 554^***^	0.540^***^		−.328
P3 latency (Pz)	−0.365^*^	−0.495^**^	−.328	

Correlation is significant at the level ^***^ *p* < 0.0005, ^**^ *p* < 0.005, ^*^ *p* < 0.025 (2-tailed). Pearson’s correlation was used, and sample size is (*n* = 30)

As it is cleared from the behavioral results, that the HA group correctly recalled 10.89 % more information than did the LA group (see Table 1) in the memory recall task. Also, the P3 amplitude and latency analysis showed significant differences at Pz site. In addition, the P3 amplitude (Pz) and latency (Pz) bears a moderate correlation with the memory recall score as shown in Table 2. The main effect for cognitive ability (HA and LA) on both memory recall and P3 component (at Pz) was determined by employing one way MANOVA, F(3,26) = 11.267, Wilk’s Λ = 0.435, *p* < 0.0005, partial η^2^ = 0.565. The univariate analysis showed statistically significant differences between HA and LA groups in memory recall score, F(1,28) = 8.342, *p*-value = 0.007; P3 amplitude at Pz site, F(1,28) = 13.672, *p*-value = 0.001; and P3 latency at Pz site, F(1,28) = 19.531, *p*-value = 0.0005.

**Fig. 6:**
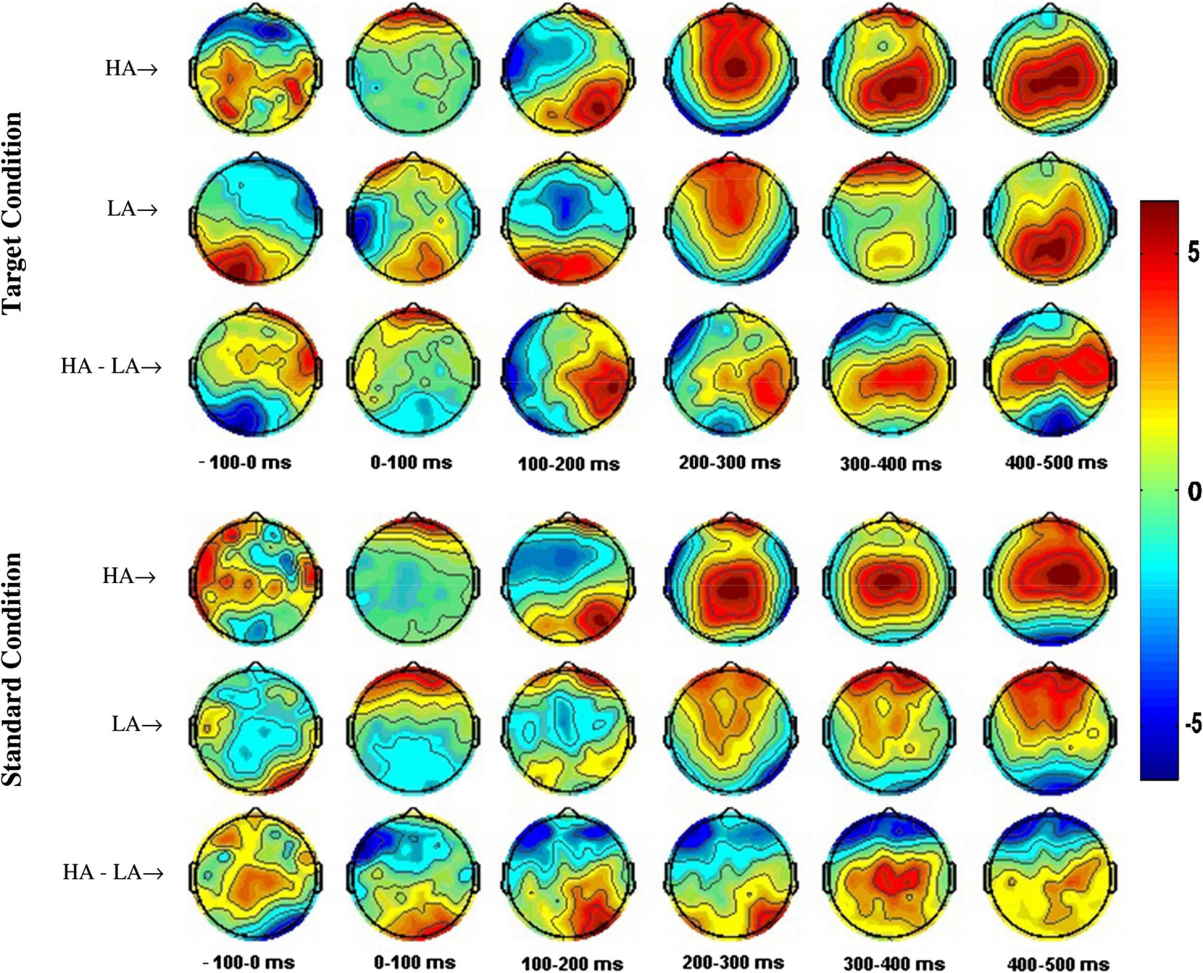
Grand average ERP responses of HA and LA Groups from 128 scalp locations. Topographic maps of mean amplitudes averaged over a 100 ms time window from −100 to 0 (pre-stimulus) and 0 to 500 ms (post-stimulus) period for visual oddball task. The first three rows of topographic maps represents brain responses to Target Stimulus for HA, LA and HA-LA; the 4th–6th row shows the brain activity in response to the Standard Stimulus for HA, LA and HA-LA, respectively

Topographic maps in Fig. 6 show scalp distributions for target vs. standard stimuli between groups as averages over a 100 ms time window from 0–500 ms (post-stimulus period). The illustrated results clearly demonstrate the differences of brain activation in the HA and LA group, particularly in the 300–400 ms and 400–500 ms time windows, the strength of activation at the *centro*-*parietal* region of the HA group was higher than the LA group, confirming the strength of the P3 component in high ability subjects.

### Prediction results

**Fig. 7:**
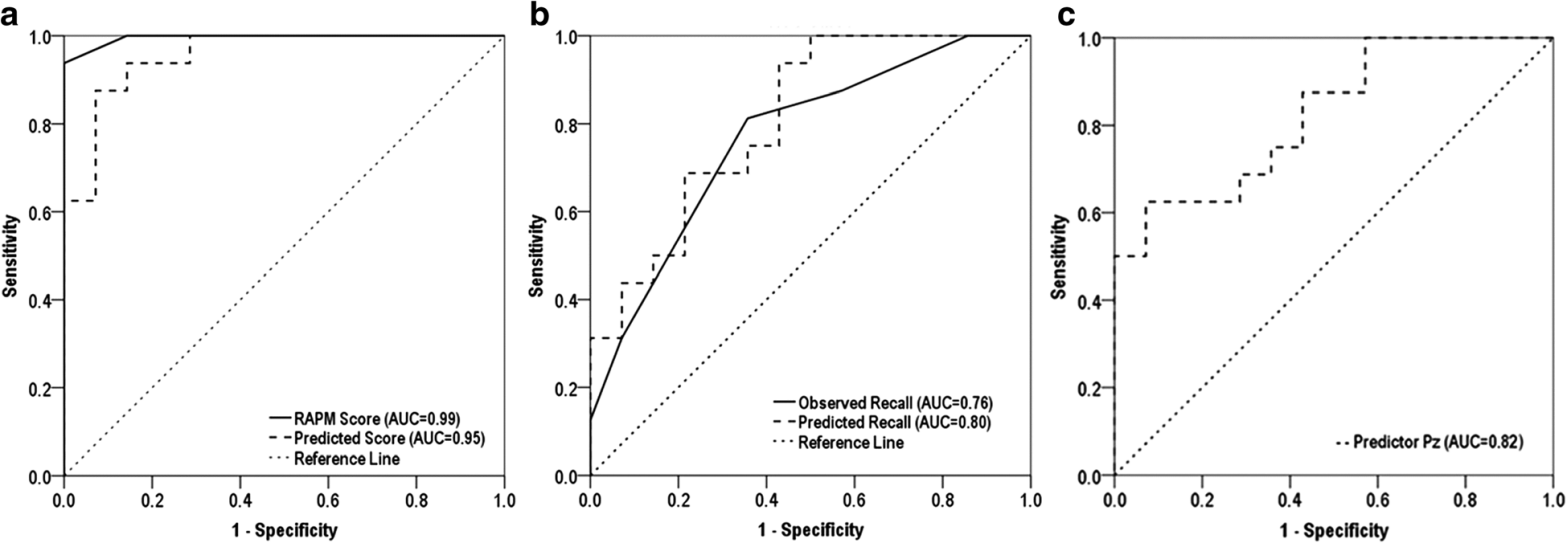
Receiver Operating Curve (ROC), (**a**) ROC for predicted and observed values of RAPM test, (**b**) ROC for predicted and observed values of memory recall, and (**c**) ROC of predictor Pz amplitude for HA and LA groups

To predict the memory recall from P3 component, multiple regression analysis with stepwise method was performed on selected 8 electrodes for P3 amplitude and latency of Pz site. The P3 amplitude (Pz) was retained in the model as predictor while rest of the electrodes was dropped due to very minimum contribution in the overall model. The P3 amplitude at Pz site statistically significantly predicted the memory recall scores, F(1,28) = 12.42, *p* = 0.001, R = 0.554 and R^2^ = 0.307. The ROC curve of the predictor (P3 amplitude at Pz) shows the AUC = 0.826, the observed and predicted recall ROC shows AUC values 0.76 and 0.80 for the HA and LA groups, respectively as illustrated in Fig. 7. In addition, the cross validated squared correlation coefficient *R*_*cv*_^2^ value = 0.255 was computed with leave-one-out (LOO) cross validation.

Similarly, multiple regression analysis with stepwise method was performed on selected 8 electrodes for P3 amplitude and latency for prediction of fluid intelligence. In the same way as in memory recall, the model retained Pz amplitude for prediction. The P3 amplitude at Pz site statistically significantly predicted the fluid intelligence (RAPM scores), F(1,28) = 11.54, *p* = 0.002, R = 0.540 and R^2^ = 0.292. The cross validated squared correlation coefficient *R*_*cv*_^2^ value = 0.225 was computed with leave-one-out (LOO) cross validation. The ROC curve shows AUC = 0.99 for observed RAPM score and AUC = 0.95 for predicted values of RAPM (see, Fig. 7). These results indicate very good diagnosis of HA and LA groups using predicted values of RAPM.

#### Verification of regression assumptions

**Fig. 8:**
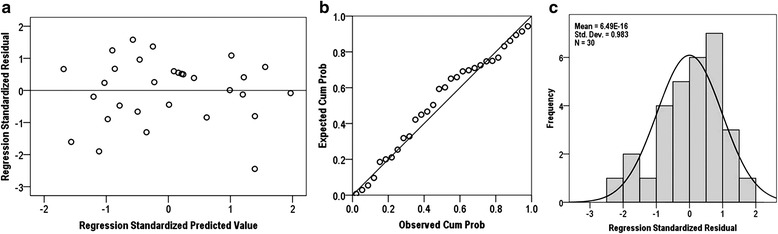
**a** Scatter plot of regression standardized residual against the regression standardized predicted value of dependent variable (Memory recall). **b** Normal P-P plot of regression standardized residual, the plot of residual fit the expected pattern well enough to support the claim that the residual is normally distributed. **c** Normal distribution plot of regression standardized residual with zero mean value and unit variance (approx.). Hence, verified the regression assumptions for memory recall, i.e., normality, linearity and homoscedasticity

Regression analysis for prediction of memory recall, the mean value of the residual is about 6.49 × 10^−16^, i.e., very close to zero. Thus, the first regression assumption is verified. Figure 8a-c, presents a normal distribution for the standardized residual which is the verification of the second assumption. The scatter plot of the residual against the predicted variable shows no specific pattern to be observed, hence verifying the third assumption (constant variance) and the fourth assumption (independence).

**Fig. 9:**
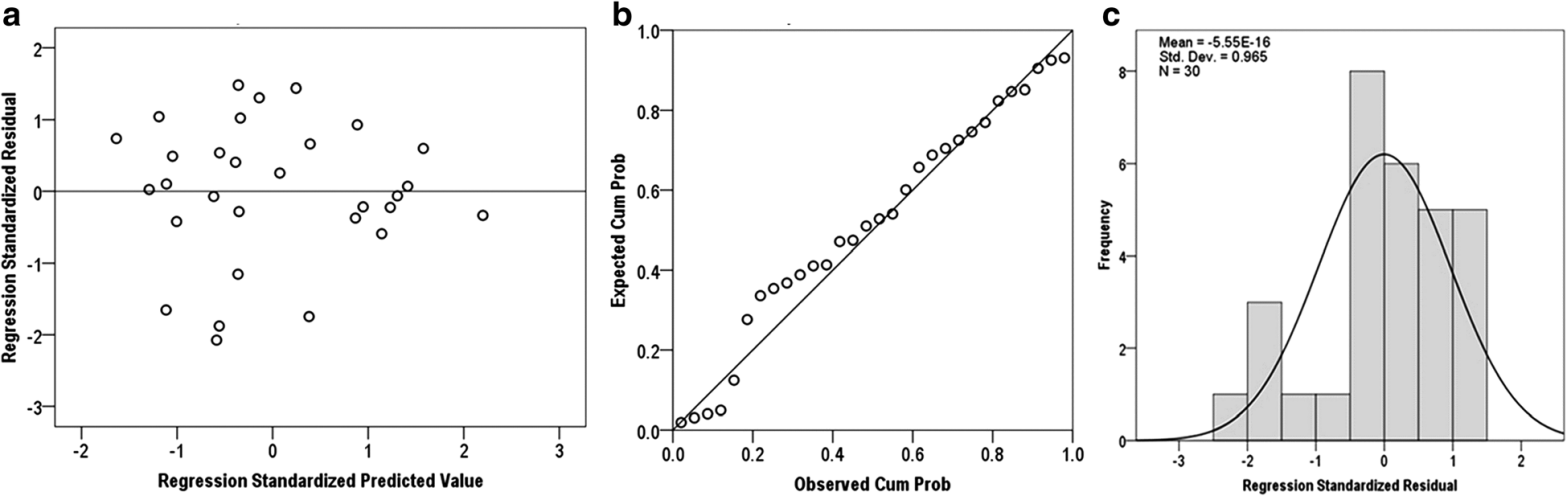
**a** Scatter plot of regression standardized residual against of regression standardized predicted value of dependent variable (RAPM Score). **b** Normal P-P plot of regression standardized residual, the plot of residual fit the expected pattern well enough to support the claim that the residual is normally distributed. **c** Normal distribution plot of regression standardized residual with zero mean value and unit variance (approx.). These plots verified the regression assumptions for fluid intelligence, i.e., normality, linearity and homoscedasticity

Similarly, regression analysis for prediction of fluid intelligence, the mean value of the residual is about −5.55 × 10^−16^, i.e., very close to zero. Figure 9a-c shows normal distribution plot and the scatter plot, which does not show any specific pattern. The scatter plot of standardized residuals against predicted values presents a random pattern centered around the line of zero standard residual value. So, there is no clear relationship between the residual and the predicted values. Thus, the regression model assumptions are considered and verified.

## Discussion

### Behavioral findings

There are significant differences in ACC and RT of oddball and memory recall task between groups. However, the overall ACC of target detection was 75 % in the oddball task in this study. The ACC was relatively reduced compared to previous studies, e.g., Kiehi et al., [33] reported 96 % target accuracy, Stevens et al., [34] 97.8 % target accuracy and Brazdil et al., [35] found 99 % target accuracy. However, these studies have used longer ITI duration (0.75 to 1.5 s) and short distance from the display monitor (60–70 cm). Furthermore, previous studies that reported ceiling accuracy in the 2-stimulus oddball task, had used ‘O’ and ‘X’ characters or string of these characters as standard and target stimuli [34–36]; while this study used ‘box’ and ‘sphere’ shapes of same size as stimuli. These may be the reasons of reduced accuracy reported in this study. However, further work may be needed to investigate the factors that may influence the target detection accuracy, such as stimulus duration, visual stimulus types and size, ITI interval, and distance of the display monitor from participant’s setting position.

### ERP findings

We investigated the relationship of ERPs to learning and memory performance as well as fluid intelligence. In support of our hypothesis, what now follows is a detailed discussion on the mutual relationship of fluid intelligence, learning and memory performance, and the P3 component.

First, an association between fluid intelligence with learning and memory performance is discussed as revealed in the educational psychology literature revisited in this study. Second, findings of a positive relationship between fluid intelligence and the P3 component are described. Third, the association of P3 with learning and memory performance is also discussed, representing the main objective of this study and contribution to the literature.

#### Relationship of fluid intelligence with learning & memory performance

This study investigated the association of fluid intelligence with learning and memory performance. Performance metrics as calculated from the learning and memory tasks are highly sensitive to RAPM (see Table 1). In this regard, our findings accord with common results from prior studies showing that individual differences in fluid intelligence are strongly associated with learning and memory abilities. These prior studies reported that intelligence is a valid predictor of educational performance [2, 6, 37, 38]. Our findings are comparable with studies documenting the relationship of cognitive ability with educational achievement [6], school performance [2], and academic achievement [37, 38]. However, most of these studies assessed educational performance as either indicated by grade point average (GPA) or via simple task metrics for learning and memory such as basic number skills, spelling, word-reading and artificial grammar skills. By contrast, we used a more complex learning approach related to scientific concepts (human anatomy) for which participants had no or very little knowledge. In general, the present study essentially validates the claim that performance efficiency (high accuracy and speed) is a critical dimension of mental ability; i.e., highly cognitive individuals tend to perform faster and respond more accurately to fluid cognitive challenges as well as learning and memory recall tasks.

#### The relationship of fluid intelligence with P3

Performance metrics as computed from the oddball task challenge strongly reflect cognitive ability as shown by RAPM scores (Table 1). Correct and speedy responses were higher for HA subjects compared to LA subjects. A positive correlation between P3 amplitude with RAPM and a negative correlation between P3 latency and RAPM at parietal site (Pz) was observed. Furthermore, P3 amplitude strength and latency during the 300–500 ms time window for the parietal region differentiated both groups (see: Table 3 and Fig. 6). The P3 amplitude at Pz site statistically significantly predicted the RAPM score, which shows link between the P3 and fluid intelligence (see, Fig. 8). P3 amplitude is sensitive to the actual volume of attentional resources that are engaged while the CNS manipulates a stimulus, which appears to have aided the discrimination of ‘target’ from ‘standard’ stimuli during the oddball challenge [9]. These particular results are also consistent with previously reported findings by referees [7, 17, 39], where an auditory oddball task was used. Again, these studies also reported that high intelligence subjects produced larger P3 peaks with short latency at the parietal locus, as validated by the present study (see Table 3); the only difference being the cited studies used an auditory oddball task whereas we used the visual oddball task. Hence, our findings clearly validate prior observations which posited that larger P3 peaks at centro-parietal loci, accompanied by short latency at parietal loci, are both associated with learning & memory performances irrespective of stimulus type.

**Table 3:** Average amplitude and latency of P300 components

Features	High ability	Low ability	Effect size (Cohen’s d)
Amplitude			
P3	3.60 (1.7)	3.61 (1.7)	0.01
P4	3.02 (1.3)	3.16 (1.3)	0.11
O1	3.40 (1.3)	3.46 (1.5)	0.04
O2	3.39 (1.7)	3.07 (1.6)	0.20
Fz	4.24 (1.9)	3.86 (1.8)	0.21
Cz	3.31 (1.2)	2.85 (1.0)	0.43
^ *^Pz	4.76 (1.0)	3.48 (0.8)	1.45
Oz	3.55 (1.7)	3.42 (2.1)	0.07
Latency			
P3	400.37 (50.8)	428.71 (65.8)	0.49
P4	390.12 (49.0)	399.71 (70.0)	0.17
O1	352.25 (66.2)	397.42 (70.0)	0.69
O2	346.62 (70.0)	351.57 (69.6)	0.07
Fz	334.25 (52.6)	365.85 (76.5)	0.50
Cz	383.87 (69.2)	411.42 (68.5)	0.41
^ *^Pz	378.62 (32.4)	436.28 (30.7)	1.68
Oz	340.62 (68.9)	388.00 (54.6)	0.78

Note: Results are organized as mean plus (standard deviation) in 2^nd^ and 3rd columns

*Indicates a significant difference between groups: *t*-test *p-*value <0.025)

#### Relationship of P3 with learning and memory performance

Previous ERP studies investigated the brain in ERP memory based tasks such as semantic memory tasks [40], episodic memory (EM effect) tasks [41], and recognition memory tasks [42]. These studies linked the P3 component with the memory processes directly. The memory processes including encoding of stimulus, retention, and recollection/retrieval of stimulus had been associated with the P3 component, especially the midline electrodes position and the centro-parietal regions (for review see, [42–44]). In the process of memory encoding (sematic encoding―a word or picture), the left inferior prefrontal cortex had been reported to be important for successful encoding. Also, the negative ERP activity (negative current density) over left inferior frontal scalp between 410 and 800 ms corresponds well to regions of activation. This had been confirmed by fMRI studies for both episodic and semantic memory retrieval task (see for review, [43]). Similarly, in recognition memory, familiarity and recollection process had been supported by ERPs studies [42]. The ‘parietal’ and ‘mid-frontal’ old/new effects in the recognition memory based ERP studies had been reported as sensitive to variations in the memory performance and the strength of source of memory [45, 46]. In the neurological prospective, the oddball sequence is compared to the previous stimulus and when a new stimulus is processed the system engages the attentional mechanisms to update the neural representation of the stimulus context and the P3 is elicited [47, 48].

In the context of indirect association of ERPs with learning & memory, we observed that the amplitude of P3 is positively correlated and that the latency of P3 is negatively correlated with learning and memory abilities at the parietal site (see Tables 2 & Fig. 5). Low P3 amplitude at the parietal region of the LA group, compared to the HA group in the 300–400 ms time window, reflects low attentional resource allocation for the LA group which was further confirmed by their lower scores for learning and memory.

The P3 amplitude at Pz site statistically significantly predicted the learning & memory performance, which shows link between the P3 and learning & memory ability. In brief, P3 amplitude strength at the parietal region, especially in the 300–400 and 400–500 ms time windows, reflects ability for learning and memory recall. These results, therefore, may have potential implications for academic practice in learning institutions concerning (i) the evaluation of a candidate’s cognitive ability for selection in a particular academic program; (ii) use as evidence for the implementation of certain teaching-learning strategies; and (iii) use as a screening tool for the recruitment of new candidates for training and educational purposes.

#### Limitations of the study

The present study has few limitations which are important to consider during future studies on this subject. One of the limitations is that we used 2-stimulus oddball task, which only produced P3b peak. However, the P3a component (3-stimulus oddball) may be helpful in predicting the learning & memory ability. In addition, due to unavailability of Advanced Progressive Matrices Norm for university students of different nationality, the raw RAPM score was used instead of converting to IQ score. The sample size is only 30 subjects and the P3 amplitude at Pz site may show better prediction if recorded from large sample, because the P3 amplitude at Pz is also larger as compared to other regions (see, Fig. 9). Further, due to small sample size the present findings are not enough to claim that P3 amplitude alone can predict the learning and memory recall performance. However, future studies can be conducted to explore the validity of learning & memory ability prediction by P3 component. In addition, this study investigated the relationship of P3 with learning & memory ability for young adults only. Finally, the learning material used in this study was related to human anatomy & physiology contents; thus the P3 may not be generalized to link with learning ability of all types of academic learning contents or memory recall ability. High density EEG system such as 128-channels used in this study may not be suitable for just recording few electrodes EEG.

## Conclusion

The variations in parietal region of the P3 component of ERP are associated with cognitive ability and that subjects who scored higher in RAPM trials produced higher EEG scalp activity in these regions. In fact, the P3 amplitude at the centro-parietal regions are associated with an individual’s learning and memory abilities. Variations in attentional engagement, perception and information processing for both groups during the oddball challenge reflected fluid intelligence and further indicated a relationship between P3 and learning and memory abilities.

Further studies could be undertaken to validate the P300 component as a predictor for academic learning with a larger and highly analogous group of individuals.
